# Recent Advances in Experimental Whole Genome Haplotyping Methods

**DOI:** 10.3390/ijms18091944

**Published:** 2017-09-11

**Authors:** Mengting Huang, Jing Tu, Zuhong Lu

**Affiliations:** State Key Lab of Bioelectronics, School of Biological Science and Medical Engineering, Southeast University, Nanjing 210096, China; 220174577@seu.edu.cn (M.H.); jtu@seu.edu.cn (J.T.)

**Keywords:** haplotype, haplotyping, phasing, next generation sequencing

## Abstract

Haplotype plays a vital role in diverse fields; however, the sequencing technologies cannot resolve haplotype directly. Pioneers demonstrated several approaches to resolve haplotype in the early years, which was extensively reviewed. Since then, numerous methods have been developed recently that have significantly improved phasing performance. Here, we review experimental methods that have emerged mainly over the past five years, and categorize them into five classes according to their maximum scale of contiguity: (i) encapsulation, (ii) 3D structure capture and construction, (iii) compartmentalization, (iv) fluorography, (v) long-read sequencing. Several subsections of certain methods are attached to each class as instances. We also discuss the relative advantages and disadvantages of different classes and make comparisons among representative methods of each class.

## 1. Introduction

Haplotyping has been a crucial issue in genetic research and clinical medicine over the past decades [1,2,3]. In genetics, haplotypes refer to the sequences of genetic variants that belong to a single chromosome. The process of assigning variants to corresponding haplotypes is termed phasing or haplotyping. Although the diploid nature of human genomes was discovered more than 50 years ago [4], researchers had not been aware of the significance of the haplotype until DNA sequencing was widely applied. Haplotypes can provide more information than unphased genotypes in diverse fields, such as identifying genotype-phenotype associations [3,5,6], exploring pharmacology and genetic diseases [7,8,9], and elucidating population structure and histories [10,11,12,13].

In the early stages, assisted by chromosomal fluorescence in situ hybridization (FISH) or long-range polymerase chain reaction (PCR), only targeted haplotyping of specific haploid loci was achievable [1,14,15]. The exploitation of large-insert clones by bacterial artificial chromosomes (BACs) enabled the Human Genome Project [16,17] to contain extensive haplotype information. The first phased personal diploid genome, known as HuRef, also adopted BAC and mate-paired Sanger sequencing reads [18]. With the advent of next-generation sequencing (NGS), the decreasing cost and soaring throughput makes this a cost-effective approach for haplotyping. However, the short reads of NGS find it difficult to cover more than one heterozygous variant, which makes it difficult for them to assist phasing. Only if heterozygote variants were covered within one read or a pair of reads could haplotype linkage be constructed. Even facilitated by paired-end libraries, the maximum length of linkage is only 3.5 kb [19]. To overcome this limitation, several experimental techniques have been developed. Although some inferential methods can estimate haplotypes based on population data or pedigree, they were elaborately reviewed before [20]. Moreover, to fully and accurately phase genomes, the assistance of experimental methods is inevitable.

Here, we mainly review the experimental methods developed in the past five years, and evaluate their relative advantages and disadvantages. According to their maximum linkage range from large to small, we categorize them into five classes: (i) encapsulation, (ii) 3D structure capture and construction, (iii) compartmentalization, (iv) fluorography, (v) long-read sequencing. Each class is named according to its principle. In encapsulation methods, chromosomes are packaged into haploid units to obtain haplotype information. The methods of 3D structure capture and construction construct linkage between two distant genomic loci by the structure of chromosome. In compartmentalization, long intact DNA fragments are compartmentalized into massive parallel pools by limiting dilution. Fluorography uses fluorescent dye to label SNPs, which are then imaged by high resolution fluorescence microscopy. The methods of long-read sequencing include some innovated sequencing approaches that are able to directly phase haplotype by their long reads. Several subsections of certain methods are attached to each class as an example. Although some new sequencing strategies, such as genome mapping and long-read sequencing, cannot resolve haplotypes independently, they are included because of their novelty.

## 2. Encapsulation

As the human chromosomes naturally encapsulate haploid homologues, directly sequencing DNA of separate chromosomes can be a straightforward way to generate haplotypes. Methods based on encapsulation make use of the packaged haploid information in single chromosomes. Some of them separate homologous chromosomes using sophisticated devices [21,22,23,24] or the procedure of meiosis [25,26,27]. Another approach developed recently differentiates template strands of sister chromatids during mitosis [28]. All these methods can obtain chromosome-length haplotypes before DNA extraction, but they are restricted by their need for specialized instruments, laborious experimental operation or the requirement for an intact single cell.

### 2.1. Chromosomes Separation

In the early stages, exploiting encapsulated haplotype mainly focused on artificially separating homologous chromosomes. Considering the minute size of a chromosome, sophisticated devices were usually required. Laser capture microdissection [21], microfluidic sorting devices [22], and FACS-mediated single chromosome sorting [23] were designed in succession to sort the desired chromosomes. Once the separated chromosomes are harvested, PCR or multiple displacement amplification (MDA) is inevitable in consideration of the trace content of DNA.

After meiosis, the human gamete contains only one set of homologous chromosomes, which can be an ideal material for studying haplotypes. However, due to technological challenges of performing single-cell genome analysis [29,30,31], whole genome sequencing of single gametes was not fully achieved until 2012. Wang et al. [24] reformed their microfluidic chromosome sorting device [22] and performed parallel analysis of many individual sperm. Although bias and errors of amplification were introduced by MDA, the limited reaction volume in each microfluidic channel reduced them, thus mitigating the problem. Lu et al. [25] used Multiple Annealing and Looping Based Amplification Cycles (MALBAC) to amplify DNA extracted from single sperm. The MALBAC technique was reported to exhibit a higher uniformity of genome coverage than MDA [32]. Hou et al. [27] also used MALBAC-based sequencing technology to phase genomes by single human oocytes. Oocytes, which require invasive surgery to extract, are more challenging to retrieve than sperm cells. For all the above-described methods, the uneven uniformity still limits the scale of haplotypes. In most cases, haplotypes obtained from human gametes were incomplete unless they are sequenced deeper or acquired by other assisting methods. Moreover, meiotic recombination can result in false phasing of the somatic genome. Although this could be resolved by sequencing massive single gametes in parallel, extra DNA library construction would increase the cost.

### 2.2. Single-Cell DNA Template Strand Sequencing

**Figure 1:**
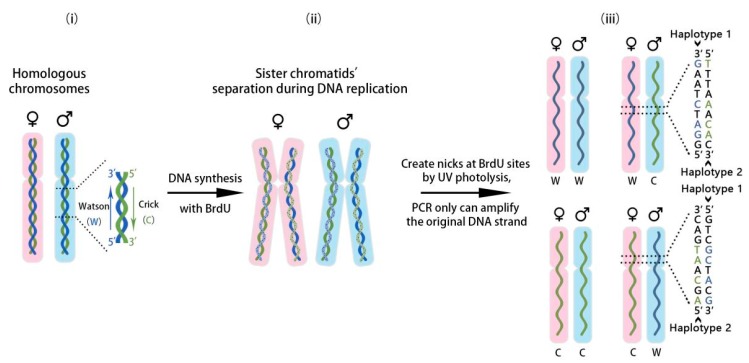
The experimental pipeline of single-cell template strand sequencing (Strand-seq) [28]. (**i**) Two homologous chromosomes, one maternal chromosome (light pink) and one paternal chromosome (light blue), are shown. Each chromosome contains a Crick template strand (green curve) and a Watson template strand (blue curve); (**ii**) During DNA replication, hemi-substituted sister chromatids, both of which contain one BrdU-positive synthesized strand (spotted curve) and one BrdU-negative template strand (solid curve), are generated in the presence of BrdU; (**iii**) Four cases are presented after segregation of sister chromatids. The BrdU-positive strands are selectively removed during library construction; thus, only the original template DNA strands (solid curve) are sequenced. When both Crick and Watson template strands are inherited, different parental homologs can be identified from their orientation. The examples of possible sequences for haplotyping (haplotype 1 and haplotype 2) are demonstrated in detail.

Single-cell DNA template strand sequencing (Strand-seq) was first reported by Falconer et al. [33] to map DNA rearrangements at high resolution. This method achieves identification of template strands of sister chromatids during DNA replication. When it was applied in haplotyping by Porubský et al. [28] in 2016, the encapsulated haploid information within the template strands could be acquired independently. In genetics, the Watson Strand (W; the blue strand in Figure 1i) refers to a 5′ to 3′ strand, whereas the Crick Strand (C; the green strand in Figure 1i) refers to a strand with the opposite orientation [34]. To perform Strand-seq, cells are cultured with BrdU for one round of DNA replication during mitosis and then harvested. Sister chromatids duplicated from the same chromosome both contain hemi-substituted genomic DNA (the mixed strands of DNA with one solid curve and one dotted curve in Figure 1ii). UV photolysis is applied to create nicks on the BrdU-positive strand, hence the newly synthesized strand cannot be amplified by the indexed primers during the PCR process. As the BrdU incorporated strand is removed after PCR, there will be only four types of production; two Watson templates (WW), two Crick template strands (CC), or a combination of Watson and Crick templates (WC) (Figure 1iii). By identifying which strand of the indices was sequenced, the result can be distinguished by the read count of each strand after single-cell sequencing. Only the type of a combination of Watson and Crick templates is useful for phasing. In this case, the Watson Strand and the Crick Strand, which represent different parental homologs, can be identified by their orientation. Haploid reads generated by indexed Illumina sequencing can be phased into chromosome-length haplotypes, even spanning sequence gaps, centromeres, and regions of homozygosity. However, to encompass all genomic single nucleotide variants (SNVs), more than one hundred single-cell libraries would need to be constructed. Furthermore, other data, such as regular WGS data, is required to mitigate the influence of low genome coverage.

## 3. 3D Structure Capture and Construction

DNA is not only the unidimensional sequence that provides information about heredity and variation. The 3D structure of DNA in chromosomes may contain more physical and biological information. The crosslinking between protein and DNA forms proximity ligation. Two distant parts in linear DNA can be very close to each other when twined and folded into a chromosome. Capturing linkages that contains more than one SNP locus has the potential to determine their haploid relationships. In most cases, the two linked parts belong to a homologous chromosome, because linkage mostly happens intra-chromosomally instead of inter-chromosomally [35,36]. Capturing chromosome conformation (3C) [37] and related methods, such as 3C combined with sequencing or 3C-on-chip (4C) [38,39], are techniques for identifying chromosomal interactions. High-resolution chromosome conformation capture (Hi-C) [40] is an advanced method derived from 3C and 4C, which is also used for whole genome haplotyping, now. By exploiting the 3D structure of DNA, capturing chromosome interactions in vivo and artificially constructing sub-chromatin structure in vitro have the potential to generate chromosome-spanning haplotypes.

### 3.1. 3D Structure Capture In Vivo

**Figure 2:**
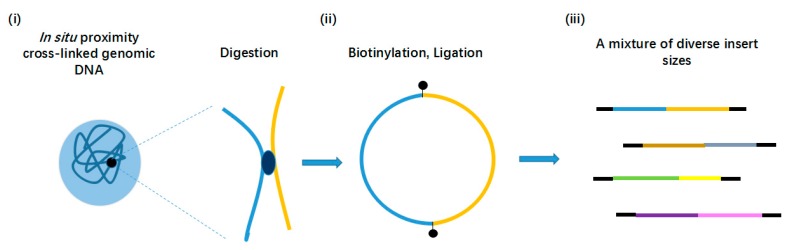
Experimental pipeline of proximity-ligation [35]. (**i**) The cross-linked DNA is digested with a restriction enzyme; (**ii**) The resulting sticky ends are filled in with biotinylated nucleotides and ligated to create chimeric loops; (**iii**) Biotinylated junctions are isolated with streptavidin beads. Consequently, the paired-end library contains fragments of diverse insert sizes, which span between 500 bp and chromosome length.

Selvaraj et al. [35] performed proximity-ligation by Hi-C protocol to reconstruct whole-genome haplotypes in vivo in 2013, which is termed HaploSeq. The cross-linked DNA was digested with a restriction enzyme and then looped together to preserve the linkage. After DNA library construction and shotgun sequencing, the proximity-ligation reads (Figure 2) help consolidate the small local haplotype blocks (built from conventional short-insert sequencing reads). These blocks ultimately phased ~81% of alleles from 17× sequencing [35]. Vree et al. [41] also exploited the 3D property of chromosomes to target re-sequencing and haplotyping genomic regions. Connecting linearly distant DNA is the key point for Hi-C libraries to generate large-scale haplotype blocks. However, this kind of connection mainly results from the nucleosome-wound DNA fiber instead of from the whole chromosome. Conversely, the complex structure of chromosomes in nuclei contains many confounding signals, which may interfere with the phasing. For instance, telomeres are often connected in nuclei [42]. Furthermore, the position of linkage in vivo and the density of heterozygous variants seriously influences the resolution of haplotypes [35].

### 3.2. 3D Structure Construction In Vitro

**Figure 3:**
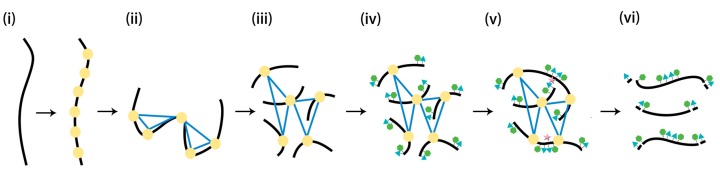
Construction protocol of “Chicago” library [42]. (**i**) The purified histones (light yellow) bind DNA (black curve) to reconstitute chromatin in vitro; (**ii**) Formaldehyde fixes chromatin and forms crosslinks (blue lines); (**iii**) Fixed chromatin is digested with restriction enzyme and generates sticky ends; (**iv**) Free sticky ends are filled in with thiolated (green hexagons) and biotinylated (blue triangles) nucleotides; (**v**) Blunt ends are ligated and the points of ligations are indicated by pink five-pointed stars; (**vi**) The proteins and the terminal biotinylated nucleotides are removed but the interior sequences are protected by the thiolated nucleotides to construct the “Chicago” library.

Compared with capturing the chromatin interactions in vivo, artificially reconstituting chromatin in vitro may have a higher resolution and signal-noise ratio (SNR). In 2016, Putnam et al. [42] demonstrated an approach, “Chicago”, to reconstitute DNA long-rang linkage in vitro. The extracted DNA was assembled into chromatin by chromatin assembly factors and purified histones. Then standard Hi-C protocol was applied to the artificial chromatin to capture the linkage (Figure 3). With the help of this approach, the noise rate was approximately one spurious link between an unrelated 500 kb genomic windows, and haploid reads ranging from 10 kb to 150 kb were 99.83% consistent with the standard. “Chicago” addresses the limitation that interactions only happen in “chromosome territories”. It extends the region where the linkage happens, which helps generate comprehensive haplotype blocks. However, both “Chicago” and the Hi-C method still have a limitation. The heterozygous variants far from restriction enzyme cut sites are seldom sequenced, which means that it always needs the help of other methods to phase the whole genome.

## 4. Compartmentalization

Separating homologous DNA from its heterogenous part is the primary means of haplotyping. The higher the purity that the extracted homologous sequences have, the better the quality the phasing can access. Under this precondition, the dilution pools strategy was initially brought up by Li et al. [43] to study single diploid cells and single sperm. Dear and Cook [44] then demonstrated the general approach, and Burgtorf et al. [45] and Raymond et al. [46] refined it. With this approach, limiting dilution makes compartmentalizes long, intact DNA fragments into massive parallel pools. Based on Poisson Distribution, there are only a few or no genomic DNA fragments divided into each pool. The possibility of heterogenous fragments appearing in the same pool is poor. The sequenced reads of each pool are tracked by barcodes, sorted into sub-haploid units, and assembled into small homologous blocks. Although methods based on compartmentalization do not need specialized instruments or complex experimental operations, constructing massive DNA libraries makes them challenging to commercialize. Recently, several works have been reported to address this challenge by virtual compartments [47] or automatically barcoded library construction [48]. 

### 4.1. Traditional Pool-Based Haplotyping

Peters et al. [49] demonstrated Long Fragment Read (LFR) technology for haplotyping in 2012. Long parental DNA fragments were stochastically separated into physically distinct pools to create sub-haploid compartments. The input DNA was only about 100 pg per sample. Instead of exploiting fosmid clones like the previous studies [50,51,52], MDA was used as a uniform approach of whole genome amplification. As a result, 92% of the heterozygous SNPs, on average, were phased into long contigs with N50s of ~1 Mb and ~500 kb, respectively, in two samples, which means that 50% of haplotype-resolved sequences (by length) were within blocks of at least ~1 Mb and ~500 kb. Ciotlos et al. [53] applied commercialized LFR technology to deeply analyze the highly aneuploid BT-474 cell line. Kaper et al. [54] also applied MDA in a dilution strategy, and phased more than 95% of heterozygous SNPs of a diploid genome. Apart from MDA, Kuleshov et al. [55] used long-range PCR as an amplification approach, and phased up to 99% of all SNVs. However, the trace content of DNA in each sub-haploid compartment still influences the uniformity and accuracy of amplification. Moreover, the single library preparation of each compartment makes the traditional pool-based strategy labor-intensive and costly.

### 4.2. Haplotyping Based on Contiguity-Preserving Transposition (CPT-Seq)

**Figure 4:**
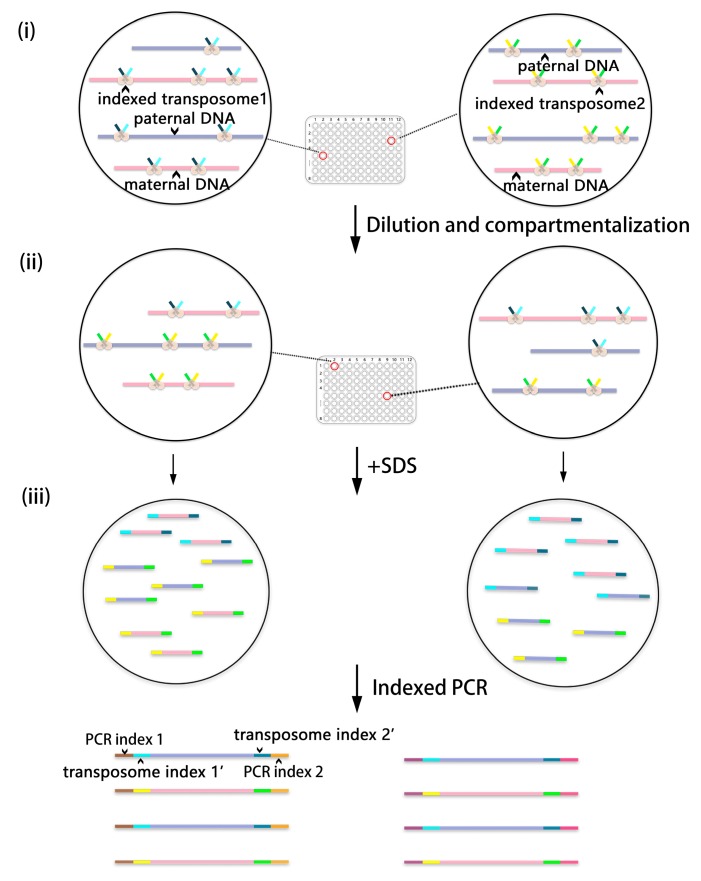
The workflow of the contiguity-preserving transposition sequencing (CPT-seq) [47]. (**i**) The maternal DNA (pink lines) and paternal DNA (purple lines) are barcoded by uniquely indexed transposon; (**ii**) The indexed libraries are pooled, diluted and redistributed into other physical compartments; (**iii**) Indexed PCR incorporates a second compartmental index into the fragments of each compartment before sequencing.

In order to decrease the cost of DNA library construction after compartmentalization, Amini et al. [47] introduced an approach in 2014 to constitute virtual compartments based on Tn5 transposition. This kind of transposition has been confirmed to bind to DNA after introducing adaptors to a DNA substrate. SDS is then added to remove the transposase, but the contiguity of target DNA and adaptors is preserved. Combined with indexed PCR, the barcoded compartments are multiplexed, but the quantity of DNA libraries does not increase. For instance, *m* = 96 compartments within maternal and paternal DNA are firstly barcoded by uniquely indexed transposon adaptors. These adaptorized libraries are then pooled, diluted and redistributed into another *n* = 96 physical compartments. Each compartment contains the DNA mixed from *m* = 96 virtual partitions. Indexed PCR incorporates a second compartmental index (*n* = 96) into each compartment. Two dimensions of indices result in a total of *m* × *n* = 96 × 96 = 9216 virtual compartments, but the number of DNA libraries remains *n* = 96 (Figure 4). The haploid information can be phased after decoding of the combinatorial indices. This strategy is quite rapid (processing time < 3 h), cost-effective and scalable. The utility of virtual compartments can be augmented when increasing the value of *m* and *n*. Nevertheless, only DNA ligated with different adaptors during transposition can be amplified during PCR, which results in a 50% loss of the DNA sample. The non-uniformity of transposition also results in amplification preference of shorter elements during PCR. Despite these shortcomings, the aggregate coverage is more than enough to compensate for the low coverage of strobed reads.

### 4.3. Linked-Read Sequencing 

**Figure 5:**
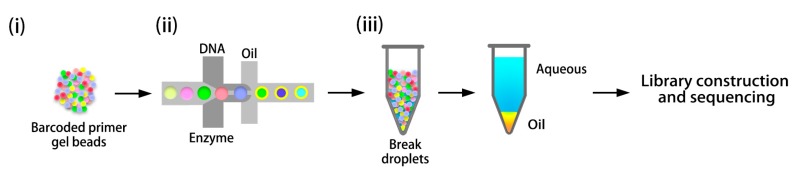
Overview of experimental process for generating linked reads [48]. (**i**) Barcoded primers are delivered by gel beads; (**ii**) Gel beads are mixed with DNA and enzymes, and then delivered to oil-surfactant solutions; (**iii**) Droplets are dissolved to release the barcoded oligonucleotides. DNA in aqueous solution is then purified and prepared to construct libraries for sequencing.

In 2016, Zheng et al. [48] presented a linked-read sequencing approach based on microfluidics, which can generate haplotype-resolved genome sequences using only nanograms of input DNA. Specifically, the barcoded primers are delivered using gel beads (Figure 5i) through microfluidic channels to a “double-cross” junction. Gel beads are incorporated here with the sample and reagent mixture, and then transformed into droplets (Figure 5ii). All the droplets will be transferred to a 96-well plate and dissolved to release the barcoded oligonucleotides (Figure 5iii). After a modified library has been prepared, standard Illumina short-read sequencing is conducted to acquire barcoded reads. Linked-read means that sequences with the same barcode have a high possibility of being duplicated from the same DNA fragment, thus being in the same haploid genome. Zheng et al. [48] verified the reliability of this approach on several genomes and phased more than 95% of SNVs with phased block N50 ranging from 0.8 Mb to 2.8Mb. Mostovoy et al. [56] combined this method with genome maps and Illumina reads, which extended phased block N50 to 4.7 Mb. This approach provides a scalable barcoded haplotype sequencing using extremely limited input DNA. The compatibility with standard downstream NGS assays gives linked-read sequencing great potential for commercialization. Conversely, this also results in biases in GC-rich regions due to the nonuniformity of Illumina sequencing [57].

Although CPT-seq and linked-read sequencing share almost the same principle for resolving haplotype, they adopt particular means to achieve compartmentalization. Thus, the requirement of the input and the performance of phasing are different. The comparison between them is shown in the Table 1.

## 5. Fluorography

The development of microscopy and fluorescent technology makes it possible to visualize nanometer-scale molecules. Methods based on fluorography use fluorescent dye to label SNPs, and high-resolution fluorescence microscopy to image them. Physical DNA imaging can span more than one SNP locus across a long DNA fragment, which is useful to phase haploid blocks. Without library construction or conventional DNA sequencing, the haplotype identification is able to be more accurate and less biased. However, none of these methods can phase the whole genome haplotype independently; while some focus on targeted haplotyping sequencing [58,59,60,61,62], others provide a genome-wide framework for phasing [56,63].

### 5.1. Targeted Fluorescence Hybridization

Under some circumstances, only part of the genome region requires determination of haplotype. Compared to retrieving the desired part from the whole genome haplotype, selectively identifying the alleles into local haplotypes is more cost-effective. Xiao et al. [58] first reported a molecular haplotyping method for labeling DNA molecules, and imaged them with total internal reflection fluorescence (TIRF) microscopy. Then, they refined this work using probes with locked nucleic acid, which raised the labeling efficiency and extended the reaction specificity [59].

FISH is widely applied in detecting specific DNA sequences and defining spatial-temporal patterns of gene expression. Beliveau et al. [60] reformed FISH-based imaging into targeted haplotyping, and developed homologue-specific OligoPaints (HOPs). With this approach, they selected thermodynamic suitable and genomically unique probe sequences that span at least one SNP on the target region. HOP probes are artificial DNA oligonucleotides that are synthesized according to the probe sequences. HOP probes are designed in pairs to distinguish SNP variants. For each oligo of a HOP probe set, a cognate oligo can be found on the same locus which differs only by the SNP variant(s). Haplotypes can be inferred from combination of hybridized HOP probes at different loci in a chromosome. Although all of them are in pairs, the SNVs are inserted into sequences to distinguish them. Haplotypes can be inferred when partner HOP probes target the same region on different homologous DNA. Beliveau et al. [60] verified this approach by examining several haploid regions, and demonstrated that higher resolution could be achieved when combined with DNA-based point accumulation for imaging in nanoscale topography (DNA-PAINT) [64] or stochastic optical reconstruction microscopy (STORM) [65].

### 5.2. Genome Mapping by Nanochannel Arrays

**Figure 6:**
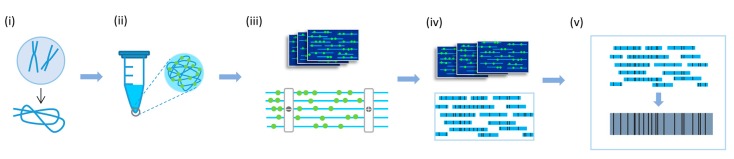
The workflow of whole genome haplotyping using genome mapping data [62]. (**i**) The high-molecular weight (HMW) DNA is extracted from the genome; (**ii**) DNA is nicked with nicking endonuclease and then labeled with fluorescent dye; (**iii**) Electrophoresis assists DNA to be loaded into the nanochannel arrays; (**iv**) Single molecule maps are assembled into consensus maps using software tools developed at BioNano Genomics; (**v**) The consensus maps from the same parental chromosome constitute a haplotype.

Combining fluorography with microfluidics, Das et al. [66] demonstrated a fluorescent labeling strategy that identifies the region of specific sequences along the stretched DNA molecules. This method was first used to detect structural variants in the human genome. In 2012, Lam et al. [61] optimized it for general use, and the method generated high-resolution sequencing motif physical maps, known as “genome maps”. After being fluorescently labeled at specific sites, long DNA molecules are stretched in nanochannel arrays. As genome maps constituted by this approach are extremely long in length, it is useful for long-range phasing (Figure 6). Cao et al. [62] used genome maps to help determine haplotypes of some hyper-variable regions. Although nanochannel arrays cannot resolve the haplotype alone, the performance of phasing is raised dramatically when it is combined with other methods. Pendleton et al. [63] phased HapMap sample NA12878 by combining nanochannel arrays, single-molecule real-time (SMRT) sequencing and Illumina short-read sequencing. The final phase block N50 reached 145 kb. Mostovoy et al. [56] utilized the data from genome maps, “Linked-Read” and Illumina reads. A better phase result was obtained, as phase block N50 raised to 4.7 Mb. Mak et al. [67] detected whole-genome structural variation by nanochannel arrays. In their work, local phasing (>150 kb regions) was routine, as DNA molecules from parental chromosomes are examined separately.

## 6. Long-Read Sequencing

Next-generation sequencing (NGS) technology is widely applied, now, due to its high speed, high throughput, high accuracy and low cost. However, the short reads of NGS (<150 bp) have difficulty covering more than one heterozygous variant, which is unlikely to resolve haplotype directly. Many experimental and computational methods have been reported to build long-range linkage of short reads to mitigate this limitation. The advent of long-read sequencing may fundamentally solve this problem. Long read length of a single DNA molecule can generate data that is directly phasable. Single-molecule real-time (SMRT) sequencing [68] and nanopore sequencing [69] are the most promising sequencing technologies that could generate long reads for haplotyping. However, both of them are still unable to phase the whole genome independently. Other sequencing methods, such as genome mapping, are combined with them to achieve high performance.

### 6.1. Single-Molecule Real-Time (SMRT) Sequencing

First invented by Eid et al. [68] in 2009, SMRT sequencing aroused great curiosity for its capacity in single molecule sequencing and long read length. This sequencing technology based on zero-mode waveguide nanostructure arrays was commercialized by the PacBio Company. Wang et al. [70] developed the PacBio-LITS method, which leverages the cost efficiency and has the potential to benefit haplotyping. Nowadays, half of the reads generated by PacBio Sequencing Systems can exceed 20 kb, and the maximum read length reaches 60 kb [71]. But it is still challenging to fully cover sequences that contain long, repetitive segments. Since no amplification process is required, the biases of sequence coverage according to GC content are drastically alleviated [57]. Thus, particularly GC- and AT-rich genome sequences can be sequenced and phased. However, considering the accuracy and cost, whole genome haplotyping still needs the assistance of short-read next-generation data. Pendleton et al. [63] integrated SMRT technology, Illumina reads and genome maps to phase the human genome. Recently, Mangul et al. [72] demonstrated Haplotype-specific Isoform Reconstruction (HapIso) to tolerate the relatively high error-rate of data from SMRT platform. They claimed it to be the first method to reconstruct haplotype-specific isoforms from long-read sequencing.

### 6.2. Nanopore Sequencing

Nanopore sequencing is based on the concept of identifying each base of a sequence when a DNA molecule passes through nanoscale pores. The different bases or base pairs are distinguished by the change of electric current. However, the fast translocation speed of DNA is one of the major hurdles of the design [73]. Recorded signal is sometimes contributed by several nucleotides. Cherf et al. [74] and Manrao et al. [75] used polymerase to slow DNA translocation speed. Laszlo et al. [76] solved the adjacent bases signal problem by measuring and identifying ion current according to all 256 four-nucleotide combinations. Fuller et al. [77] demonstrated a nanopore-based synthesis strategy that uses four different polymer tags to differentiate nucleotides during their incorporation into a growing DNA strand. Although not all of these nanopore sequencing strategies have been applied in haplotyping, they are of great potential in generating direct data on haplotypes in the future.

## 7. Discussion and Conclusions

To fully interpret the human genome, haplotyping is an inevitable trend. Many experimental methods have been developed recently to facilitate this process. The above-described methods vary in linkage range, genome phase percentage, and experimental complexity and instrument requirements. The comparison among representative methods of each class is shown in Table 2. Methods based on encapsulation have the potential to phase chromosome-length haplotypes, but most of them need specialized instruments and skilled experimental operation. The uncertainty of the harvest may lead to massive parallel experiments, which are labor-intensive. Methods that make use of the 3D structure of chromatin build linkages between two linearly distant but spatially close DNA sequences. They can also generate chromosome-spanning haplotypes with no need for sophisticated instruments. However, the risk of false phasing inter-chromosome reads is worth noting. Compartmentalization-related methods have low system complexity, but mainly focus on the local haplotype blocks. It has previous required laborious library construction and deep sequencing, but the advent of CPT-seq and linked read mitigates the situation. Fluorography-related methods need microscopy and fluorescent dye. They provide a whole genome framework for phasing, but also require the assistance of other methods. As for long-read sequencing, it can generate long reads spanning several heterozygous variants, but the accuracy and cost performance still need improvement.

Haplotypes can provide more information than only the genotype in genetic diseases, genome association, inheritance pattern of pedigrees and populations. Methods developed in the past five years drastically accelerate the speed of resolving haplotype and improve the performance of phasing. Some innovative methods, such as nanopore sequencing, will have great potential in haplotyping once they break through the bottleneck. With the development of precision medicine and the popularization of DNA sequencing, these haplotyping methods will be broadly used in the genetic field to facilitate a deeper understanding of human genome.

## Figures and Tables

**Table 1:** Comparison of CPT-seq and Linked-read sequencing.

Haplotyping Method	CPT-Seq [47]	Linked-Read Sequencing [48]
Input DNA	Highly intact HMW ^1^ genomic DNA	300 genomic equivalents, or 1 ng of HMW genomic DNA
The number of compartments	9216 (can be extended in principle)	100,000
Genomic DNA per partition	21–62 Mb	3 Mb
The percentage of phasing SNPs	93.15–98.53%	95–99%
N50 phase block(kb)	490–2286	962.11–2834.44
false positive rate ^2^	Relatively high	Low

^1^ HMW, high-molecular weight; ^2^ the possibilities of two HMW molecules overlapping the same genomic loci but with opposing haplotypes.

**Table 2:** Comparison among representative methods of each class.

Haplotyping Method	Strand-Seq [28]	“Chicago” [42]	Linked-Read [48]	Nanochannel Arrays [62]	SMRT [68]
Attached class	Encapsulation	3D structure capture and construction	Compartmentalization	Fluorography	Long-read sequencing
Scale of contiguity	Chromosome length	Chromosome length	40–200 kb	20–220 kb	20–60 kb
Principle	Identifying sister chromatids during DNA replication	Reconstructing chromatin by Hi-C protocol	Stochastically barcoding HMW DNA molecules and creating linked-reads	Generate high-resolution physical maps of chromosomes	Sequencing long DNA fragments
Library preparation	Single-cell libraries required, but without WGA	No specific requirement	No specific requirement	No need of library construction	Specific libraries required
Instrument and reagents	BrdU reagent	Chromatin assembly kit and Hi-C related reagents	Cartridge reservoirs and barcoded primer gel beads	Irys System of Bionano Company	Sequencer based on zero-mode waveguide nanoarrays
Input DNA	BrdU incorporated DNA within single cells	HMW DNA 5.5 µg	HMW DNA 1 ng	HMW DNA	HMW DNA
Independent method or not	YES	assistance required	YES	assistance required	assistance required
Labor intensiveness	High	Moderate	Moderate	Moderate	Moderate
Cost	High ^1^	Moderate	Moderate	Moderate	High

^1^ WGS and single cell library construction costs.
